# Community Consultation for Planned Emergent Use Research: Experiences From an Academic Medical Center

**DOI:** 10.2196/10062

**Published:** 2018-05-02

**Authors:** Nathan J Smischney, Jasleen Pannu, Richard F Hinds, Jennifer B McCormick

**Affiliations:** ^1^ Department of Anesthesiology and Perioperative Medicine Division of Critical Care Medicine Mayo Clinic Rochester, MN United States; ^2^ Department of Internal Medicine Division of Pulmonary and Critical Care Medicine Mayo Clinic Rochester, MN United States; ^3^ College of Medicine Penn State University Hershey, PA United States

**Keywords:** qualitative research, research design, research ethics

## Abstract

**Background:**

Emergent use research—research involving human subjects that have a life-threatening medical condition and who are unlikely to provide informed consent—in critical illness is fraught with challenges related to obtaining informed consent. Per federal regulations, to meet criteria to conduct such trials, the investigators have to seek community consultations. Effective ways of obtaining this consultation remains ill-defined.

**Objective:**

We sought to describe methods, interpretations, and our experiences of conducting community consultation in a planned emergent use randomized controlled trial.

**Methods:**

As part of a planned emergent use clinical trial in our study, community consultation consisted of four focus groups sessions with members from the community in which the clinical trial was conducted. Three focus group sessions were conducted with members who had an affiliation to Mayo Clinic, and the other focus group session was conducted with non-Mayo affiliation members. The feedback from the focus group sessions led to the creation of the public notification plan. The public was notified of the trial through community meetings as well as social media.

**Results:**

As compared to community meetings, focus group sessions resulted in greater attendance with more interactive discussions. Moreover, focus group sessions resulted in greater in-depth conversations leading to institutional acceptance of the clinical trial under study.

**Conclusions:**

Exception from informed consent can be acceptable to the community. Focus groups provided better participation and valuable interactive insight as compared to community meetings in our study. This could serve as a valuable guide for investigators pursuing exception from informed consent in their research studies.

## Introduction

### Background

Emergent use research may be defined as research involving human subjects who have a life-threatening condition and who are unable to provide informed consent. This type of research is important as interventions carried in an emergent setting may not undergo the same degree of scientific rigor that other interventions would in a nonemergent setting due to the requirement of informed consent. Informed consent forms the basis of patient autonomy. However, obtaining informed consent during emergent use research is often not feasible because of the critical nature of potential participants’ condition. Surrogates who could provide consent on behalf of the patient are frequently not immediately present and, even if they are, the situational context is usually such that approaching the patient’s family about a research study is ethically questionable [1]. Regulations authorizing exception from informed consent for research conducted in emergency and critical care settings were approved by the Food and Drug Administration (FDA) in 1996 [2]. As part of this regulation, investigators must conduct an Institutional Review Board (IRB)–approved form of community consultation prior to the start of the study. They must also conduct community notification, the mechanism by which the local public is informed of the study. Community consultation can facilitate the building of trust while demonstrating respect within the community in which the study is to be conducted. During community consultation, the community is informed of the study prior to its launch with opportunities to give opinions about the study and engage in discussion with the investigators [3,4]. Dickert et al attempted to define the following four ethical goals of community consultation as (a) enhanced protection, (b) enhanced benefits, (c) legitimacy, and (d) shared responsibility [5]. Predefining these goals specific to each study can help organize time, objectives, and resources, both for researchers as well as IRBs.

Community consultation can be conducted in numerous ways. It has been differentiated by some into interactive and noninteractive methods [4]. Focus groups, community/town hall meetings, in-person interviews, investigator-initiated or existing group meetings comprise the interactive methods, whereas personal or Web-based surveys are examples of noninteractive methods. In a recent multicentered study by Dickert et al, interactive community consultation methods demonstrated significantly increased acceptance to participate, recall, and understand the study as compared to noninteractive methods [4]. Several other single-center studies have shown similar results [6-8]. Although interactive methods also showed significantly higher variability and lower recall of risks associated with the study, they offered a unique opportunity to explore the views and concerns of the participants. Our study entitled “Ketamine/Propofol Admixture ‘Ketofol’ at Induction in the Critically Ill Against Etomidate: KEEP PACE Clinical Trial” met FDA definitions to qualify as planned emergent use research [9]. Thereafter, as per FDA requirement, a plan for community consultation and notification was developed. Our aim was to conduct two interactive forms of community consultation (focus groups and community meetings), describe our experiences with both methods, and to demonstrate how our consultation plans led to public notification.

## Methods

### Community Consultation

We used focus groups for our community consultation, inviting participants from local communities most likely to be under investigation. We conducted focus group sessions using standard approaches [10]. Focus group participants were notified via flyers and research announcements surrounding Mayo Clinic Rochester and Olmsted Medical Center (OMC). Adults (≥ 18 years) were asked to join a focus group to share their views on consent for an upcoming clinical trial. Refreshments were served with sandwiches prepared at each session. Questions asked by the moderator centered on reasons for conducting the study, how a patient may be a potential participant, and if so, how they would feel about enrolling into a research trial without knowingly giving permission. All focus groups were led by a trained moderator (JM). We used focus groups because we believed the opportunities to explore community members’ views and concerns would be indispensable for our study, considering the critical nature of the intervention. The information received and interpreted from these discussions was then integrated and disseminated through community notification. Formal analyses performed from the focus group sessions were not performed as this was a descriptive study by nature. Additionally, as recommended by our IRB, de novo community meetings were organized in a similar manner as the focus groups and conducted to provide information and answer further questions as part of the community notification plan. Community-engaged research is typically conducted in the form of public (community) meetings at our institution. Hence, the recommendation from the local IRB. We expected to observe that of the two forms of interactive methods, focus group sessions would provide more valuable insight into community views given the smaller setting in which these were conducted.

### Community Notification

Notification included a summary of all key aspects of the study, including the nature of planned emergency research, study protocol, medications, patient participation, waiver of informed consent, and links to information about the study and the investigators. Our community notification plan was developed in accordance with the FDA regulations as well as inputs from focus group discussions (community consultations) regarding utilizing traditional and social media.

## Results

### Community Consultation

Three focus group sessions were conducted on the Rochester Mayo Clinic campus and one was conducted at OMC facility. OMC is the other primary healthcare provider in Rochester besides Mayo Clinic. It has a smaller research arm, and OMC patients usually do not have a direct Mayo affiliation (employment, spousal employment, and retiree) and thus are likely not to participate in the study. In total, we had 27 participants in these sessions; 11, 6, 5 and 5 in each session respectively. It was clear to the investigators that participants involved in the focus group session at OMC were more skeptical of research than the three focus group sessions done at Mayo Clinic. Participants from all focus groups were supportive of the study and understood that obtaining informed consent was not practical or “feasible” for the study. Focus group members concurred that study participants and/or surrogates “need to be informed of study participation.” There was a general consensus that the “exact timing” and approach for notifying the subjects would need to be “contextual and determined on a case-by-case basis.”

In our trial, after the acute event has passed and when time is considered appropriate, our study coordinator informs the patient or the surrogate verbally as well as in written content about the patients’ enrollment. In the event the patient is deceased, the surrogate is approached through the same process, but within a 48-hour window from the time of death. A consistent theme among focus group members was also the need for research subjects or surrogates to be “able to withdraw their participation by having their data not used in the study.” Interestingly, this theme was not brought forward by the moderating team, but rather it was spontaneously raised by the participants. With further exploration, focus group participants expressed that “data removal,” even after the fact, “was congruent and one in the same as the participant undergoing informed consent.” Because this was a substantial theme and is consistent with local values of the community, we have incorporated this into our study design. An opt-out mechanism through development of “community wide opt out registry” was thought to be “neither necessary nor helpful” by our focus groups.

Unfortunately, we did not have meaningful attendance at our community meetings. Thus, our notification plan relied on the feedback from the focus group sessions.

### Community Notification

Our focus group participants generally agreed that the plan of publishing information about the study in the local newspaper was valuable; however, several other papers and newsletters with local circulation were suggested. Participants also agreed that making radio announcements were a good idea, but suggested considering regularly scheduled radio shows as well as utilizing ethnic radio and television stations available in our community to reach immigrant populations, who may not be fluent in English. Notices of our study are being published in the area’s largest daily newspaper approximately every 2 weeks and in local circulars with a different target audience. In the current trial, our first public notification meeting had no attendees. To broaden the public access to our study information, a YouTube video about 9 minutes in length was created [11]. To date, the video has been viewed 481 times. Another strategy utilized during our trial was to provide the same video through our hospitals “on-demand” video system and post information in our intensive care units and their corresponding waiting rooms, allowing patients and family members to access the video and receive information about the trial.

## Discussion

### Focus Groups

Feedback obtained from our focus group sessions played a pivotal role in formulating the plan for prospective waiver of consent, later retrospective collection of consent, and the public notification plan. In particular, the feedback on data withdrawal after enrollment into the trial was extremely valuable as data withdrawal after the fact is not supported by federal regulations and thus was paramount in formulating our consent plan. Furthermore, the timing of enrollment notification was invaluable and served to establish the time period on when to approach patients or their surrogates about trial enrollment. This is an important milestone to strive for when community consultations are performed and failure to acknowledge community concern is not desirable. We conducted four focus group sessions. Prior literature indicates that conducting overly extensive consultation may obstruct important work versus insufficient consultation that can be ineffective [5]. Given the recurring themes with each session and no new themes during our last session, we felt that further consultation would likely be ineffective.

### Community Meetings

Interestingly, we were not able to generate meaningful attendance from participants in our investigator-initiated community meetings. Although the community meetings were part of the community notification plan in our study, they can be included within community consultation, as both involve participation from the community, whether seeking advice or consent. To our knowledge, a comparison of efficacy between such community meetings and focus groups is not currently found in the literature. The difference in response is probably multifactorial, depending more on regional factors or incentives offered. In addition, this observation may be explained by the fact that these were de novo meetings and not current existing meetings for the public. Targeting existing community group meetings showed a good attendance in a recent multicenter study [4].

### Institutional Review Board

Throughout the process of conducting and planning this particular trial, we faced several challenges from the local IRB. Currently, our institution has never served as primary sponsor of a planned emergent-use research study. The reservations our IRB had regarding planned emergent use research centered on patient autonomy. The IRB felt that without patient autonomy, the trial was unethical and therefore requested an alternative to prospective informed consent; thus leading to the creation of data withdrawal for those subjects who later declined participation in the trial. Retrospective consent is obtained when the data is already established. This form of consent allows, to a degree, patient autonomy. However, this is in direct conflict with the FDA and their requirements for studies conducted under planned emergent use research. The removal of data could potentially bias the study results as those most likely to die may request that their data be removed due to interventions labeled as research, more so than those who had a favorable outcome. Following several meetings with both regulatory boards, we allowed data removal on study procedures that were to be performed after talking to the patient and/or legally authorized representative. However, data obtained up to that point is considered part of the research without removal. This re-enforced the context-sensitive nature of informing the participants of their enrollment into the study.

### Limitations

In our study, we attempted to capture a diverse range of opinions through attempts to incorporate views of subjects that are not the target population and most likely to have reservations against participation in research (OMC participants). These inclusions by all means could not have been complete. A quantitative analysis defining level of trust, understanding, and acceptance of participation was not performed. This can be an important prospect for future studies as there are currently limited empirical data on the effectiveness of consultation strategies.

### Conclusions

Choosing an appropriate community consultation method for exception from informed consent continues to pose a challenge for the research community and the IRB. Lack of definite benchmarks and knowledge about the kind of methods best suited for each kind of study warrant research in this area. In these circumstances, it may be beneficial to recommend the presence of IRB members at such sessions. This would help illustrate efficacy of each method to ensure autonomy and respect of study subjects and would also improve the dynamics of understanding between research teams and IRB. For example, having an IRB member present during discussions regarding data use/removal would have streamlined the process and would have demonstrated to the institutional body the community views on patient autonomy. We have attempted to describe our experience with obtaining and interpreting community consultations as well as conducting community notifications. We intend for these experiences to serve as a guide for novel emergent use researchers and a stepping stone for further multi-centered research studies.

### Future Directions

It would be of benefit to perform comparative studies between the different community participation events available and to delineate the efficacy of each method in relation to the kind of study being conducted. As our study posed a higher risk of death with some important decisions that were dependent on interpretations of community consults (eg, data gathering), we feel distinct interactions in focus group sessions are beneficial for studies similar to ours.

## Abbreviations

FDA: Food and Drug Administration
IRB: institutional review board
OMC: Olmsted Medical Center
